# Recent advances in the exploration and discovery of SARS-CoV-2 inhibitory peptides from edible animal proteins

**DOI:** 10.3389/fnut.2024.1346510

**Published:** 2024-02-07

**Authors:** Xiaoyue Kong, Wei Wang, Yizhi Zhong, Nan Wang, Kaiwen Bai, Yi Wu, Qianhui Qi, Yu Zhang, Xingquan Liu, Junran Xie

**Affiliations:** ^1^College of Food and Health, Zhejiang Agriculture and Forestry University, Hangzhou, China; ^2^School of Biological and Chemical Engineering, Zhejiang University of Science and Technology, Hangzhou, China; ^3^Department of Anesthesiology, Sir Run Run Shaw Hospital, School of Medicine, Zhejiang University, Hangzhou, China; ^4^College of Biology and Environmental Engineering, Zhejiang Shuren University, Hangzhou, China; ^5^Institute of Quality and Standard for Agriculture Products, Zhejiang Academy of Agricultural Science, Hangzhou, China

**Keywords:** efficacy, mechanisms, peptides derived from animal proteins, SARS-CoV-2, computer-aided design methods, drug delivery strategies

## Abstract

The severe acute respiratory syndrome coronavirus type 2 (SARS-CoV-2), which causes the coronavirus disease 2019 (COVID-19), is spreading worldwide. Although the COVID-19 epidemic has passed its peak of transmission, the harm it has caused deserves our attention. Scientists are striving to develop medications that can effectively treat COVID-19 symptoms without causing any adverse reactions. SARS-CoV-2 inhibitory peptides derived from animal proteins have a wide range of functional activities in addition to safety. Identifying animal protein sources is crucial to obtaining SARS-CoV-2 inhibitory peptides from animal sources. This review aims to reveal the mechanisms of action of these peptides on SARS-CoV-2 and the possibility of animal proteins as a material source of SARS-CoV-2 inhibitory peptides. Also, it introduces the utilization of computer-aided design methods, phage display, and drug delivery strategies in the research on SARS-CoV-2 inhibitor peptides from animal proteins. In order to identify new antiviral peptides and boost their efficiency, we recommend investigating the interaction between SARS-CoV-2 inhibitory peptides from animal protein sources and non-structural proteins (Nsps) using a variety of technologies, including computer-aided drug approaches, phage display techniques, and drug delivery techniques. This article provides useful information for the development of novel anti-COVID-19 drugs.

## 1 Introduction

In 2020, a global outbreak of coronavirus disease 2019 (COVID-19) posed a severe threat to human life and health. The pathogen has been identified as severe acute respiratory syndrome coronavirus type 2 (SARS-CoV-2), and the receptor for this pathogen has been found to be angiotensin-converting enzyme 2 (ACE2) (1). In addition, there are other factors associated with SARS-CoV-2 infection. Researchers have adopted various methods to prevent and treat COVID-19, such as by blocking the binding of ACE2 to SARS-CoV-2, or by preventing the entry of SARS-CoV-2 into the body (2). New strains of SARS-CoV-2 have appeared, and the virus is spreading more quickly. There is an urgent need for highly effective and safe antiviral medications to stop the virus from spreading and to speed up the recovery process. The development of antiviral medications against COVID-19 is also particularly important to addressing vaccine escape and treating symptoms caused by new strains. Currently available anti-COVID-19 drugs show significant efficacy, but their accessibility and price pose challenges to widespread adoption.

Bioactive peptides are small fragments of isolated proteins with potent biological effects (3), and they typically contain 2–20 amino acids (4). Bioactive peptides offer a multitude of benefits for the human body, including anti-hypertensive, anti-SARS-CoV-2 (5), anti-inflammatory (6), anti-bacterial (7, 8), anti-diabetic (9), and anti-cancer (10). Also, bioactive peptides can protect humans from SARS-CoV-2 infection (11). Animal proteins, which contain all the nine essential amino acids, can provide nutrition essential for the human body (12). Functional peptides from animal protein sources are peptides derived from animal proteins, such as muscle protein, egg protein, and milk protein. Functional peptides of animal origin have several advantages. First, they do not have any side effects; second, they do not lead to drug resistance; third, they can be easily absorbed by the human body; and fourth, they have a high level of safety; fifth, they exhibit unique biological activities. Plant-based protein ingredients often contain allergenic proteins and other anti-nutritional factors, so that may not be suitable for pregnant women, children, and allergy sufferers (13).

Animal protein-derived SARS-CoV-2 inhibitory peptides have been shown to act on ACE2 and receptor-binding domain (RBD). IRW, an animal protein-derived peptide, may inhibit the interaction of spike (S) protein of SARS-CoV-2 with ACE2 and have greater contact with RBD. Cellular assays have demonstrated that IRW can inhibit the expression of SARS-CoV-2 S protein and ACE2 (14). With the characteristics of designability, strong stability, small volume, and easy absorption, peptides from animal proteins can be combined with nanomaterials for targeted therapies. Thanks to these characteristics, peptides from animal proteins have played an important role in preventing and treating COVID-19. Zhao found that a human-derived peptide 4H30 defends against SARS-CoV-2 by preventing its entry, membrane fusion, and release. Animal experiments showed that 4H30 can decrease the concentration of the virus *in vivo* (15). Many other animal protein-derived peptides can also protect against SARS-CoV-2 besides 4H30 (16). However, most of the peptides currently used for SARS-CoV-2 prevention are of synthetic origin, leaving substantial room for the application and development of animal protein-derived peptides. Safety, designability, and high efficiency make animal protein-derived peptides a potential material for formulated foods that serve particular medical purposes. In comparison to SARS-CoV-2 inhibitors of other sources, the number of known SARS-CoV-2 inhibitory peptides from animal proteins is too small. The source materials with the greatest potential to produce SARS-CoV-2 inhibitory peptides from animal proteins have not been reported. Peptides have a short half-life, so it is important to maintain their effective concentration. The development of technology has made it feasible to combine computational biology and phage display methods with the exploration of novel potent SARS-CoV-2 inhibitory peptides from animal proteins. Additionally, the drug distribution strategy can be used to guarantee the concentration of those peptides *in vivo*.

This review aims to reveal the mechanisms of action of these animal protein-derived peptides on SARS-CoV-2 and explore the possibilities of using animal proteins as a source of SARS-CoV-2 inhibitory peptides. Also, the review introduces the application of computer-aided design methods, phage display, and drug delivery strategies in identifying or using SARS-CoV-2 inhibitory peptides from animal proteins.

## 2 Severe acute respiratory syndrome coronavirus type 2

COVID-19 showed high levels of lethality and infectivity at first. Its lethality has been decreasing as the virus mutates. Researchers have successfully determined that SARS-CoV-2 is the primary cause (17, 18). Patients experience variable degrees of discomfort within 6 months following acute COVID-19 infection (19).

### 2.1 The structure of SARS-CoV-2

**Figure 1:**
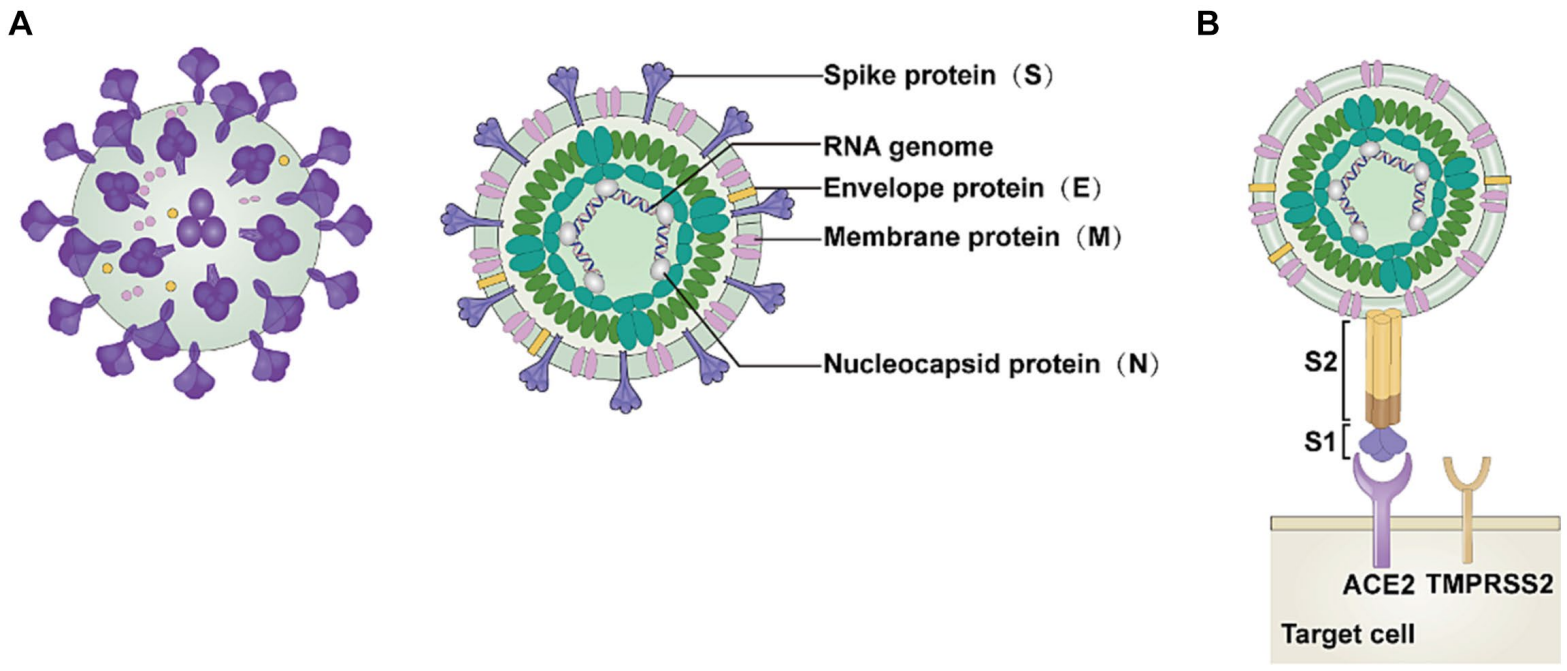
Structure of SARS-CoV-2. **(A)** The severe acute respiratory syndrome coronavirus 2 (SARS-CoV-2) virion consists of the following structural proteins: membrane protein (M), spike protein (S), nucleocapsid protein (N), and envelope protein (E). **(B)** The S protein attaches to the host cell receptor angiotensin-converting enzyme 2 (ACE2) using the S1 domain. This image refers to the cited literature, with some adjustments (20).

SARS-CoV-2 consists of four structural proteins: membrane protein (M), spike protein (S), nucleocapsid protein (N), and envelope protein (E) (Figure 1A) (20). S1 and S2 subunits make up the S protein, and ACE2 functions as a functional receptor by binding to the S1 subunit (Figure 1B) (1, 21). The carboxypeptidase structural domain located at the extracellular N-terminus of ACE2 can interact with SARS-CoV-2 (22). The four unique structural domains of the S1 subunit are the receptor-binding domain (RBD), two carboxy-terminal (C-terminal) structural domains (structural domain 1 and structural domain 2), and the amino-terminal (N-terminal) structural domain (23). The S2 subunit initiates membrane fusion and releases the viral RNA into the host cytoplasm (24).

In addition to the structural proteins mentioned above, SARS-CoV-2 also contains non-structural proteins (Nsps). The open reading frame 1a/1ab (ORF1a/ORF1ab), which makes up the great majority of the genetic information in the SARS-CoV-2 genome, can be translated into two polyproteins pp1a and pp1ab (25). pp1ab, which is involved in viral genome replication and production, can be broken down by viral proteases like main protease/3 chymotrypsin-like protease (M^pro^/3CL^pro^; M^pro^ is also known as 3CL^pro^) and papain-like protease (PL^pro^) into Nsps with different functions. There are a total of 16 types of Nsps, Nsp1-16. Nsp12, and cofactors Nsp7/8 facilitate genome replication and transcription (26).

### 2.2 Pathogenesis of SARS-CoV-2

**Figure 2:**
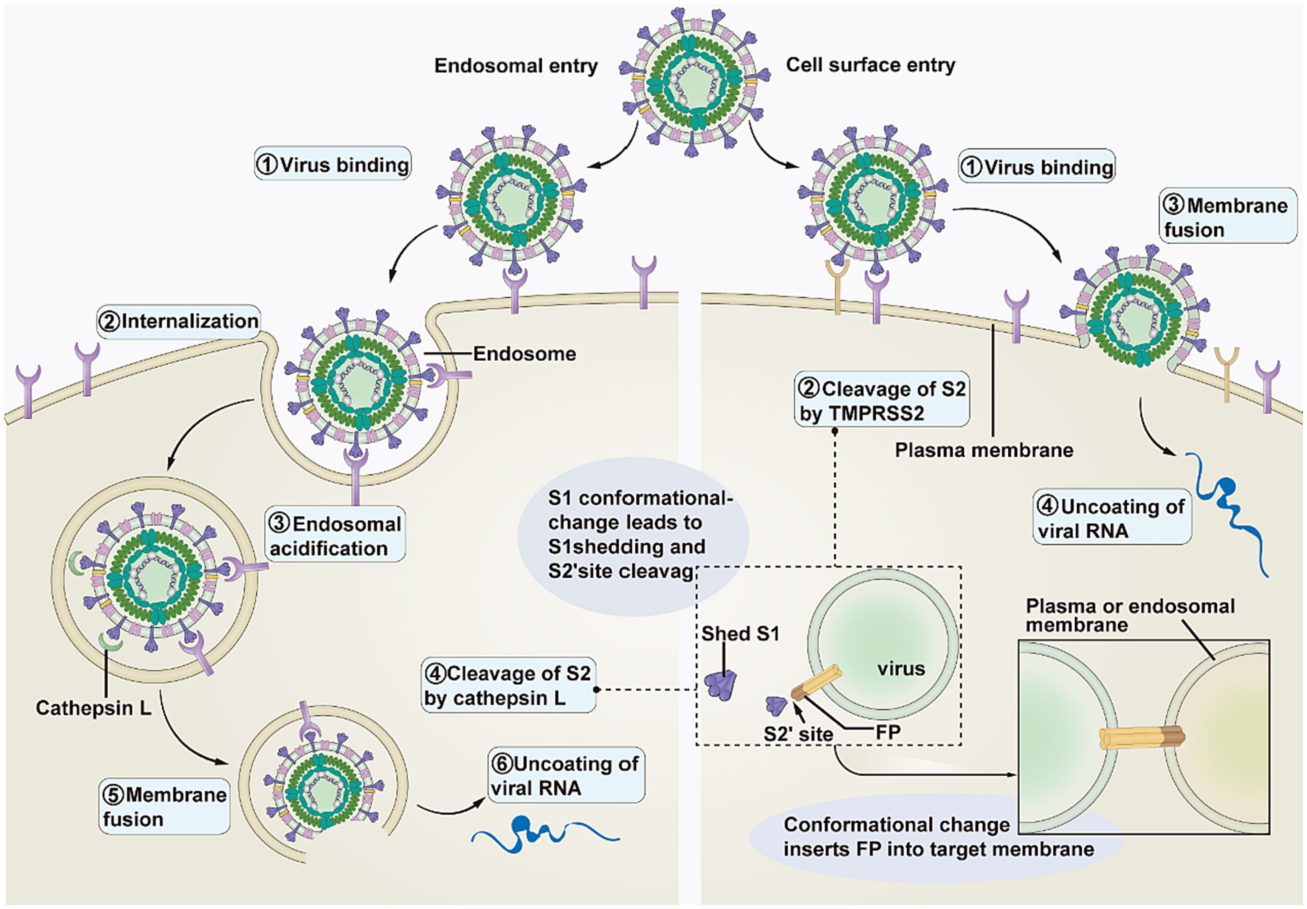
Pathways of SARS-CoV-2 entering the host cell. This image refers to Figure 3 in the cited literature, with some adjustments (23).

The SARS-CoV-2 complex enters cells through two main pathways: endocytosis and cell membrane fusion (Figure 2) (23). The transmembrane serine proteinase 2 (TMPRSS2) enables the separation of the S1 and S2 subunits (16). When little or no TMPRSS2 is present, SARS-CoV-2 enters host cells by endocytosis following endosomal acidification (27). Conversely, the SARS-CoV-2 complex enters the host cell by cell membrane fusion in the presence of a significant amount of TMPRSS2 (28).

**Figure 3:**
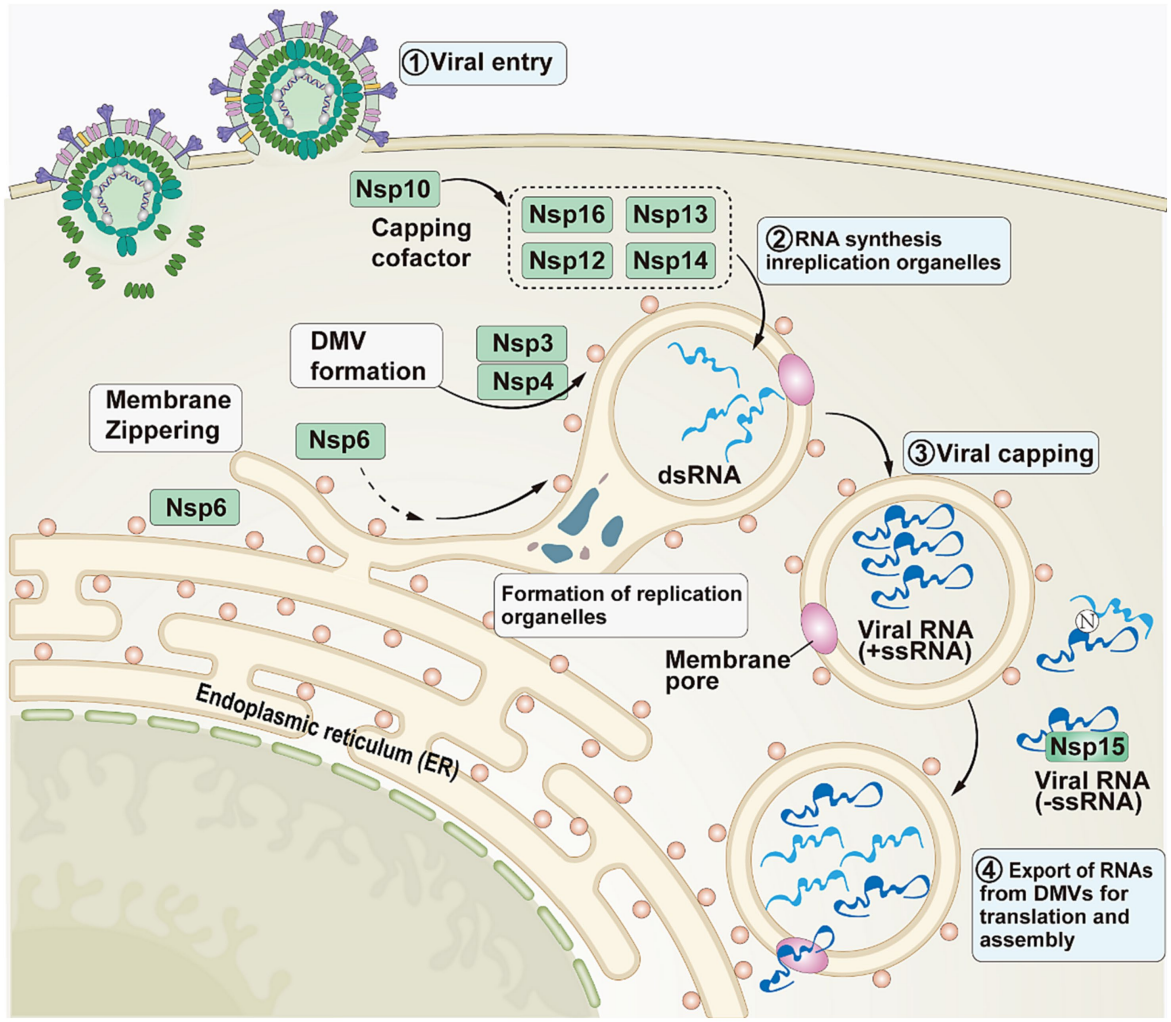
The role played by Nsps in SARS-CoV-2. This image refers to Figure 1 in the cited literature, with some adjustments (29).

In addition to the involvement in viral replication, Nsps also help SARS-CoV-2 evade host defenses (Figure 3). In the initial phase, SARS-CoV-2 enters the host cell (30). Subsequently, Nsps act as mediators in the second phase. The structural changes that Nsp3 and Nsp4 cause in the endoplasmic reticulum (ER) membrane lead to the creation of double-membrane vesicles and convoluted membranes, which are components of replication organelles (RO). To permit lipid flow, Nsp6 creates a molecular rope that resembles a zipper between the RO and the ER. In the third stage, double-stranded RNA (dsRNA) completes viral methylation through Nsp12, Nsp13, Nsp16, Nsp14, and Nsp10 (31). In order to facilitate the viral replication process without being recognized by the host, negative-sense single-stranded RNA (-ssRNA) and dsRNA are isolated from the RO. In the fourth stage, as replication intensifies, viral RNA accumulates outside the RO and is subsequently masked and/or minimized by the N protein and/or Nsp15 (29).

Therefore, ACE2 (32), S proteins (33), N proteins, TMPRSS2 (34), RBD, PL^pro^, M^pro^/3CL^pro^ (35), and Nsps are all key to blocking SARS-CoV-2.

## 3 Potential of bioactive peptides in COVID-19 treatment

Bioactive peptides have been found to play a significant role in inhibiting the activity of SARS-CoV-2. Research literature on SARS-CoV-2 has highlighted the potential inhibitory effects of whey protein peptides (36), peanut protein peptides (37), and soy cheese peptides (38). These peptides with antiviral properties offer a safer method to achieve patients’ nutritional balance and to combat viral attacks. Furthermore, these discoveries serve as a fundamental basis for the design and synthesis of antiviral drugs using peptides from animal proteins.

### 3.1 Antiviral pathways of bioactive peptides

**Figure 4:**
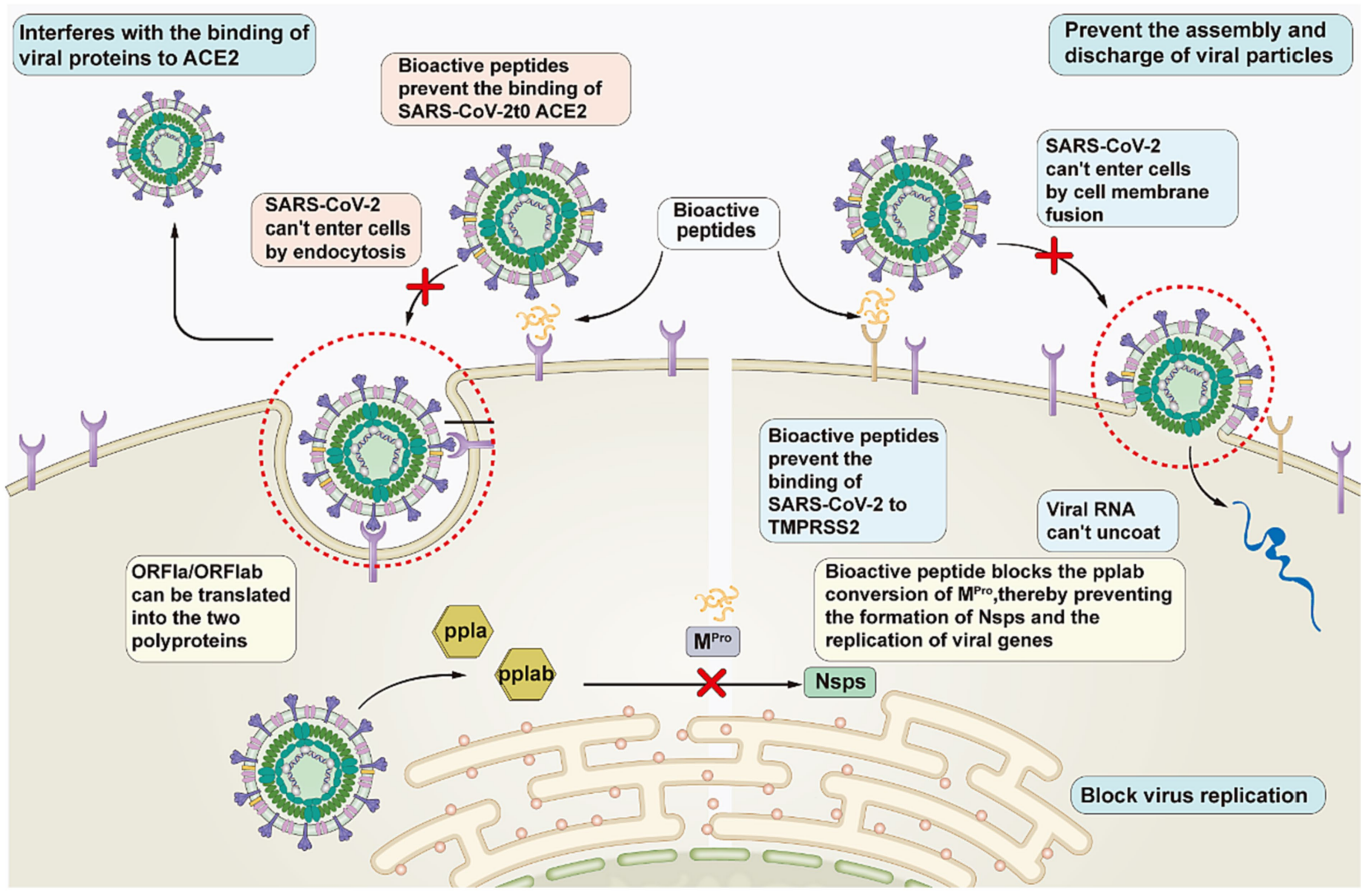
Pathways of SARS-CoV-2 inhibitory peptides.

Peptides hold great promise for the development of anti-COVID-19 drugs. Antiviral peptides can be developed based on existing knowledge of the structures and targets of viral proteins. Based on the known structure of viruses and process of infection, researchers have divided virus combating strategies into the following three categories: interfering with the binding of viral proteins to host receptors to block viral entry; blocking viral replication; and inhibiting the assembly and release of viral particles (Figure 4). (1) Interfering with the binding of viral proteins to host receptors. Peptides play a role in the prevention of COVID-19 by interfering with the binding of the SARS-CoV-2 S protein to ACE2. Curreli et al. (39) designed four peptides to block the binding of SARS-CoV-2 to ACE2 to prevent COVID-19 infection. He et al. (40) designed 15 peptides that effectively blocked the binding of ACE2 to RBD, thereby significantly reducing infection with COVID-19. (2) Blocking virus replication. The peptides stop viral replication in the body by blocking the action of PL^pro^ and M^pro^, which are involved in mediating viral replication. Liu et al. (41) described a novel peptide-drug conjugate derived from the PL^pro^-specific substrate LRGG, which has demonstrated its antiviral properties in cell experiments. (3) Preventing the assembly and discharge of viral particles. Peptides treat COVID-19 by inhibiting the activity of TMPRSS2, which encourages the assembly and release of viral particles. Shapira et al. (42) found that the peptidomimetic drug Ms-Gln-Phe-Arg-kbt inhibits TMPRSS2 activity by 83%. The effectiveness of N-0385 has been demonstrated in both cell and animal experiments.

### 3.2 Peptide vaccines

Peptides have been used in the production of vaccines. Human phase I trials have demonstrated the effectiveness of CoVac-1, a peptide-based vaccine that stimulates T-cell responses to offer long-term immunity after infection (43). Shen et al. (44) constructed an anti-COVID-19 peptide vaccine that induces human immunoglobulin G response to the RBD of SARS-CoV-2 and enhances immune responses in mice. To reduce disease severity, Eszter et al. (45) investigated T-cell-based peptide vaccines against two novel SARS-CoV-2 variants.

## 4 SARS-CoV-2 inhibitory peptides from animal protein sources

Animal products such as eggs, milk, and meat have a higher protein content and greater health benefits compared with plant-based foods. Animal peptides can be better absorbed by the human body because they have a lower molecular weight than proteins. Animal protein-derived peptides have a variety of characteristics, such as antiviral, anti-inflammatory, and antioxidant. At a time when SARS-CoV-2 poses a serious threat to human health, those peptides have become a focus in the search for a strong SARS-CoV-2 inhibitor that is free of side effects. We have summarized the SARS-CoV-2 inhibitory peptides of animal origin in Table 1.

**Table 1:** SARS-CoV-2 inhibitory peptides from animal protein sources.

Source	Production method	Target protein	Name	Peptide sequence	Effect	Reference
Human breast milk	Biochemical fractionation of milk is carried out by ion exchange chromatography	N protein and S protein	Lactoferrin (LF), mucin1 (MUC1), and α-lactalbumin (α-LA)		Reduced S-protein gene expression and N-protein gene expression in cells infected with SARS-CoV-2	(46)
Human breast milk	Biochemical fractionation of milk is carried out by ion exchange chromatography	ACE2 and S protein	α-LA		0.25 mg/mL of α-LA interferes with the interaction between S protein and ACE2	(46)
Goat milk whey	Goat milk whey was hydrolyzed by trypsin	M^pro^	β-lactoglobulin derived peptides	Ala-Leu-Pro-Met-His-Ile-Arg (ALMPHIR), Ile-Pro Ala-Val-Phe-Lys (IPAVFK)-	In molecular docking, ALPMHIR and IPAVFK are the peptides with the lowest binding energy level to M^Pro^	(47)
Goat milk whey	Goat milk whey was hydrolyzed by trypsin	S protein	β-lactoglobulin derived peptides	Ala-Leu-Pro-Met-His-Ile-Arg (ALMPHIR)	In molecular docking, ALPMHIR is the peptide with the lowest binding energy level to S protein.	(47)
Rattlesnake Venom	Rattlesnake Venom was isolated by a single cation-exchange chromatography step using a MonoS HR 10/10 column	C30 endopeptidase (3CL ^pro^)	Crotamine derivative L-peptides	D-CDP1 (KMDCRWRWKCCKK) and D-CDP7 (KMDCRWRWKSCKK)	In cellular assays, L-CDP1 and L-CDP7 inhibit SARS-CoV-2-infected cells by 80% at concentrations below 2 μM	(48)
Bee venom	Bee venom peptides are characterized using HPLC and mass spectrometry	ORF1ab, S protein and nucleocapsid phosphoprotein	Melittin of bee venom	GIGAVLKV LTTGLPALIS WIKRKRQQ- amide	Treating SARS-CoV-2 infected cells with bee venom peptide down-regulates S protein and ORF1ab	(49)
Tuna	Enzymatic hydrolysis of tuna proteins is conducted using pepsin, trypsin and chymotrypsin	M^pro^ and ACE2	Tuna peptide	EEAGGATAAQIEM	E-M, M^Pro^ and ACE2 residues form hydrogen bonds and electrostatic forces in molecular docking	(50)
Ribbonfish	Enzymatic hydrolysis of ribbonfish proteins is conducted using alkaline protease	3CL^pro^	Multifunctional fish peptides	Pro-Thr-Arg (PTR)	Molecular dynamics simulations show stable PTR binding to the 3CL^pro^ of SARS-CoV-2	(51)
Mouse		SARS-CoV-2	β-defensin-4 peptides	NGAICWGPCP TAFRQIGNCGR FRVRCCRIR(P9R)	In mouse assays, P9R was shown to significantly inhibit viral replication in mouse lungs and increase survival rates	(52)
Frog		TMPRSS2 and S protein	Frog-defensin-derived basic peptide (FBP)	RGAHIKGRWK SRCHRF(FBP)	FBP inhibits endosomal acidification of infected cells and blocks the fusion of cells transfected with S proteins with cells transfected with ACE2.	(53)
Human		ACE2	Human β-defensin 2 (hBD-2)		hBD-2 has an IC_50_ value of 2.800 ± 0.400 μM for ACE2.	(54)

### 4.1 Milk

Milk contains abundant superior proteins, fats, minerals (calcium, magnesium, and selenium), and vitamins (riboflavin, vitamin B12, and pantothenic acid). Mammals can get both nutrients and immunity from milk. Food undergoes drastic changes during fermentation. Milk fermented with *L. paracasei* has proven to have an ACE2 inhibitory effect (55), and it also has an anti-inflammatory effect. However, the study did not clarify the mechanism of action of peptides against SARS-CoV-2.

Milk’s whey protein contains high levels of mucin, lactoferrin, and α-lactalbumin. It was discovered that α-lactalbumin stops the S protein of SARS-CoV-2 from attaching to ACE2, therefore preventing the virus from entering the host (46). Lactoferrin and α-lactalbumin have demonstrated significant efficacy in preventing infection with SARS-CoV-2. The antiviral properties of whey proteins have attracted researchers’ attention (56). β**-**lactoglobulin-derived animal protein peptides are a type of lactoferrin. Çakır et al. (47) utilized *in silico* approaches to demonstrate β-lactoglobulin-derived peptides ALMPHIR and IPAVFK can bind to M^pro^. It was found that ALMPHIR and IPAVFK may interfere with viral protease and viral binding. Therefore, β-lactoglobulin derived peptides may have antiviral properties. In conclusion, additional antiviral peptides may exist in lactoferrin and α-lactalbumin in milk. According to these results, breast milk is recommended for the development of anti-COVID-19 medications. Meanwhile, these studies have contributed to an in-depth investigation of SARS-CoV-2.

The information above indicates that milk-derived peptides are effective in stopping SARS-CoV-2 from invading the human body. In addition to mucin, lactoferrin, and α-lactalbumin, peptides derived from β-lactoglobulin from animal protein sources have also been found to exhibit anti-SARS-CoV-2 properties. Thus, it is considered that peptides prepared from other breast milk proteins may have similar characteristics. Milk’s strong nutritional value improves human health while its antiviral properties provide immune protection. However, people who are prone to allergies should still be cautious of milk consumption because it contains several allergens. Notably, when those allergens undergo enzymolysis or other degradation processes, they are much less likely to trigger allergic reactions. Utilizing milk as the primary animal protein source for the development of a SARS-CoV-2 inhibitory peptide is advantageous.

### 4.2 Venom

Approximately 15% of known creatures on earth are venomous. Venom contains peptides and enzymes that can help fight cancer and alleviate autoimmune illnesses (57). Venom serves as a source of new molecules that can be used for drug discovery and development, and other physiological purposes (58). In the process of studying snake venom-derived peptides, it was found that they contain substances that can fight against SARS-CoV-2. Eberle et al. (48) used crotamine derivative L-peptides (L-CDP) derived from rattlesnake venom to attack SARS-CoV-2. Cell experiments showed that its inhibition rate against SARS-CoV-2 was 80% when the concentration of CDP1 and CDP7 was lower than 2 μM. It has been found that both CDP1 and CDP7 possess the ability to combat SARS-CoV-2. However, their mechanism of action is still unclear.

In addition to blocking the pathway of viral entry, affecting the replication and transcription of the virus is also one of the strategies adopted to prepare antiviral inhibitors. Enayathullah et al. (49) found that bee venom peptide can effectively reduce SARS-CoV-2 activity, with a LogEC_50_ value of 2.826 corresponding to 0.656 μg. Proteomic analysis showed that when treated with bee venom peptide, SARS-CoV-2 infected cells downregulate S protein and ORF1ab. ORF1a/ORF1ab are associated with viral proteases that mediate intracellular replication and transcription after SARS-CoV-2 enters the body. Overall, the anti-viral properties of bee venom peptides have been verified.

Venom peptides have two distinct effects: hindering the multiplication of viruses that have successfully infected the host organism, and preventing SARS-CoV-2 from attaching to host receptors. Venom peptides are very attractive material candidates for therapeutic medicines because of their unique selectivity, stability, and exceptional potency in regulating their molecular targets (59). Technological advances like high-throughput screening (60) methods and computational approaches have facilitated the development of tools used to simulate the interaction between venom peptides and their molecular targets. Thanks to those technological advances, the process of identifying specific venom peptides is now less expensive and more predictable, and the research on venom peptides and their antiviral capabilities have been promoted. In the future, a significant number of venom peptides are expected to be discovered.

### 4.3 Fish

According to recent studies, the protein content of most fish exceeds 15%, or 30–50% of the daily protein intake required for humans. Fish provides 75% of the animal protein intake each day (61). Fish that live in cold water have high concentrations of essential amino acids, including arginine, glycine, and lysine. Those essential amino acids can be easily absorbed by humans and are used to cure and prevent diseases (62). Many studies have been conducted on the functional activities of fish-derived peptides, such as anti-bacterial (63), blood pressure-lowering, and anti-bacterial (64). Nevertheless, research on the anti-SARS-CoV-2 effect of fish-derived peptides has been rarely reported.

In recent years, the COVID-19 epidemic has prompted more researchers to conduct research on the anti-SARS-CoV-2 effect of fish-derived peptides. Researchers have found antiviral peptides in tuna bones. Yu et al. (50) found that when combined with M^pro^, the peptide E-M extracted from tuna protein hydrolysate can prevent the polyprotein cleavage of SARS-CoV-2. Also, they found that E-M competes with SARS-CoV-2 for ACE2 binding and shows high binding stability. E-M prevents SARS-CoV-2 from binding to ACE2, thus preventing the invasion of SARS-CoV-2 and achieving an antiviral effect.

PTR (PTR = 372.2115 Da), one of the four antiviral peptides discovered by Yathisha et al. (51), exhibits the greatest efficacy. Through molecular docking and molecular dynamics techniques, it was found that PTR from ribbonfish binds to SARS-CoV-2 3CL^pro^ by establishing hydrogen bonds with residues Lys5, Gln127, Lys137, and Gly138. When comparing the binding energies, it was found that the binding ability of PTR was consistently higher than that of remdesivir in all cases. This observation indicates that PTR possesses potential antiviral properties. Nevertheless, studies on cells and animals have not confirmed the precise mechanism of action.

Fish processing generates a large amount of waste like fish skin, bones, and offal, which accounts for more than 60% of the biomass of processed fish (65). Waste accumulation leads to environmental contamination; therefore, waste management is important for not only better resource use and greater economic value of fish but also sustainable economic growth. The finding that animal protein-derived peptides from tuna and ribbonfish can suppress SARS-CoV-2 suggests that fish is a potential raw material for the preparation of antiviral peptides. The high nutritional content and other functional properties of fish-derived peptides make them suitable candidates for nutritional supplements with antiviral properties. Products derived from fish may exhibit an inherent odor, commonly known as a fishy smell. However, this issue can be effectively addressed through the deodorization process, which involves the combined use of physical, chemical, and biological techniques (66). However, fish-derived antiviral peptides are yet to be validated *in vivo*.

### 4.4 Other sources

Defensin peptide is one of the anti-microbial peptides found in the epithelial cells of vertebrates (67). Although defensin peptides can be classified as antimicrobial peptides, they also possess extensive antiviral properties (68).

Several attempts have been made to investigate the possibility of producing antiviral peptides by using positively charged defensin peptides from animal protein sources. In a study conducted by Zhao et al. (52), researchers examined the inhibitory effects of a modified positively charged mouse β-defensin-4 peptide (P9R) on the replication of SARS-CoV-2. P9R was found to be able to bind directly to the virus and thus exert inhibitory effects on the virus. Positive charges can inhibit endosomal acidification. Consequently, P9R effectively prevented endosomal acidification of the virus in the host, which prevented viral multiplication. Based on the research result, Zhao et al. then conducted a more intensive study on defensin peptides in another study. They found that modified frog-defensin-derived basic peptide (FBP) produced similar results, that is, modified FBP was found to prevent viral replication in the lungs of hamsters infected with SARS-CoV-2 (53).

The experiments showed that the introduction of positive charges inhibits acidification in the host body, and that animal protein-derived peptides bind to SARS-CoV-2. In this way, SARS-CoV-2 entry into the host cell by endocytosis is prevented, thereby mitigating the risk of infection. Modified positively charged functional peptides of animal origin block the way of viral entry into the human body to prevent SARS-CoV-2 infection, which demonstrates the role of animal protein-derived peptides in the field of antivirals. They act as an inhibitor to interfere with the viral invasion mechanism. The interaction between SARS-CoV-2 and peptides from animal protein sources can be identified and validated using fluorescence staining microscopy alone; however, a detailed examination of the specific binding sites of these peptides and SARS-CoV-2 is needed.

Moreover, it has been found that human nasal, upper respiratory, and oral mucosa contain the host defense peptide human β-defensin 2 (hBD-2), which possesses antiviral characteristics. Through molecular docking studies, Zhang (54) found that at a concentration of 12.8 μM, the recombinant hBD-2 exhibited an inhibition rate of 80% on the S protein. This suggests that hBD-2 can play an important role in the fight against COVID-19. If the expression or structure of hBD-2 is modified by added compounds, it is expected to exhibit greater antiviral efficacy. hBD-2 can be administered from the respiratory tract to block SARS-CoV-2 and is expected to become an antiviral agent.

In summary, a variety of animal-derived defensins have anti-SARS-CoV-2 effects. Defensins derived from animals are unique from the perspective of virus binding to receptor proteins. Moreover, they are distinct in that they can inhibit endosomal acidification to stop the virus from entering the body by altering the intracellular pH through the introduction of peptides and positive charges. The broad-spectrum antiviral action of defensins challenges the established knowledge of antimicrobial peptides, thereby opening up new research possibilities. Peptides from animal proteins have a wide variety of functional characteristics. Many researchers are interested in their possible anti-SARS-CoV-2 effect.

According to most recently published literature, few raw materials that can be used to prepare SARS-CoV-2 inhibitory peptides have been identified. As far as we know, the most affordable, accessible, and safe ingredient is milk. Milk-derived SARS-CoV-2 inhibitory peptides have been validated by cellular tests and has passed molecular docking predictions, thereby offering some theoretical support. Milk may be an important material source for the preparation of SARS-CoV-2 inhibitory peptides from animal protein sources. A small number of animal protein sources are available for the preparation of SARS-CoV-2 inhibitory peptides so far. Fewer studies have investigated the inhibition of viral replication; most literature focuses on blocking ACE2 or stopping ACE2 from binding to RBD. No research has been done on the relationship between animal protein-derived SARS-CoV-2 inhibitory peptides and Nsps. Nsps not only contribute to the spread of viruses but also enable viruses to evade the immune system, which needs to be tackled with in the battle against SARS-CoV-2. Research on SARS-CoV-2 inhibitory peptides of animal origin has been included in some papers on animal studies, cell analysis, bioavailability, and simulated digestion. Additionally, the relationship between Nsps and SARS-CoV-2 inhibitory peptides is unclear. Overall, SARS-CoV-2 inhibitory peptides derived from animal proteins have not been thoroughly investigated.

More in-depth research places new and higher demands on cost, raw materials, time, and error toleration. By using computational biology, the search for SARS-CoV-2 inhibitory peptides of animal origin can be carried out on a broader basis and from a higher perspective. Meanwhile, it helps reduce waste of time, money, and raw materials and increase error toleration.

## 5 Development of computational biology for SARS-CoV-2 inhibitory peptides from animal protein sources

Research on animal protein derived peptides involves preparation, extraction, separation, purification, and finally *in vitro* and *in vivo* experiments to verify the mechanism of action. With the development of science and technology, most of the results mentioned above can be obtained by employing computational biology based on known peptide sequences, enzymes, etc.

### 5.1 Computer-aided drug design methods

Computer-aided drug design applies computer software and chemical simulation techniques to predict drug-target interactions, discover novel drugs, and evaluate drug safety (69). As a computer-aided drug design approach, the bioinformatics approach can be used to perform virtual digestion and virtual screening, so as to obtain the target peptide, predict its efficacy, and search for the site of action. The overall workflow of computer-aided drug design in the research on SARS-CoV-2 inhibitory peptides of animal origin is as follows (70).

#### 5.1.1 Bioinformatics approaches

Currently, there are different kinds of peptide databases, such as the antihypertensive peptide database AHTPDB (71), the bioactive peptide database BIOPEP-UWM (72), the food protein database FeptideDB (73), the milk database MBPDB and MilkAMP (74), the fermented food database FermFooDB (75), and the insect antimicrobial peptides database APD3 (76). These databases offer useful tools and reliable sources of information for subsequent prediction of the physicochemical characteristics of peptides. Nevertheless, some of the most recently identified peptides might not be included in the databases due to information incompleteness. The creation of those databases facilitates subsequent molecular modeling and quantitative structure–activity relationships (QSAR) modeling.

Computer-simulated digestion can simulate the digestive reactions of peptides. Simulated digestion predicts theoretically generated peptides based on the potential biological activity of peptides released from proteins in the presence of specific enzymes. First, the sequences of the proteins involved are obtained from Universal Protein (Uniprot) and National Center for Biotechnology Information (NCBI). Next, software programs or web servers, like PeptideCutter, FeptideDB, Peptide Cutter, and BIOPEP-UWM, are used to simulate digestion. Villadóniga et al. (77) used computer-simulated gastrointestinal digestion to determine the stability of peptides produced from enzymatically digested α-lactalbumin in the gastrointestinal tract. However, computer simulation is still not perfect. Computer simulation outcomes may differ from the results obtained from real tests. Computer simulation represents ideal settings. In real tests, some temperature and pH values will change since real test conditions are not exactly the same as simulation conditions.

In addition to simulating gastrointestinal digestion, Web servers can be used to predict absorption, distribution, metabolism, excretion, and toxicity (ADMET). Web servers include Discovery Studio (DS) 2017 (78), admetSAR (79), etc. Chen et al. (78) isolated and purified peptides from eggs and then used databases to screen for ACE-inhibitory peptides. ADMET prediction was then performed using admetSAR to select peptides that met the ADMET criteria for further study. Bioinformatics approaches can be used to investigate the properties of novel bioactive peptides. PeptideRanker scores and ranks bioactive peptides based on their binding structure–activity relationships, with the score ranging from 0 to 0.8. Specifically, 0 represents the lowest bioactivity while 0.8 is the highest. For example, Wei et al. (80) scored 16 functional peptides found in fermented cheese using PeptideRanker, and all scored higher than 0.4. Relative molecular mass, isoelectric point, net charge, hydrophobicity index, enzyme-cleaved electron protein, and other parameters can be calculated using Expasy-PI/MW, PepDraw (81), and ExPASy-Peptidecutter (82). Peptide targets can be predicted using SwissTarget Prediction (83).

Virtual screening methods mainly fall into two categories: the ligand-based approach and the structure-based approach. Ligand-based methods, such as the QSAR method, focus on peptides that are already known to be biologically active. On the other hand, structure-based methods, like molecular docking, rely on the knowledge of the ligand structure and the receptor structure (70).

#### 5.1.2 Quantitative structure–activity relationships

Computer-aided drug design involves the utilization of quantitative structure–activity relationships (QSAR). QSAR correlates predictions based on chemical data and linear or nonlinear correlations between the observed values of molecular properties or biological activities and the calculated values of molecular structures (84).

The science of teaching computers to think and behave like humans, known as artificial intelligence (AI), is to help computers learn automatically based on real-world facts and knowledge (85). The key to realizing AI is machine learning (ML), which is a data-accessing subset of AI. ML uses data access and trend analysis to create actionable insights. Moreover, ML involves techniques like random forests, support vector machines, deep learning, neural networks, matrix, etc. (86).

Conventional QSAR models are limited to a single kind of prediction. These days, QSAR models are being used in combination with AI approaches. To improve prediction performance, most QSAR models combine multitasking templates with AI technologies. Their neural network-based techniques can simultaneously predict related targets in the same or different species as well as the same target under diverse experimental conditions (87). Grant (88) used neural networks in conjunction with cryo-electron microscopy pictures to capture molecular motions and comprehend the dynamics of proteins. Utilizing ML scoring matrices, comparative sequencing, and phylogenetic trees, four SARS-CoV-2 inhibitory peptides were found to block Mpro activity (89). Elend et al. (90) improved the quantity and caliber of Mpro inhibitors found through SARS-CoV-2 screening by training a neural network model and evolutionary algorithms on a library of drug-like chemicals.

#### 5.1.3 Molecular docking and molecular dynamics simulation

Molecular docking criteria for the evaluation of ligand-receptor binding modes are based on conformational sampling and high/low binding affinity scoring functions (91). During docking, a superior ligand is selected by sampling various locations, orientations, and conformations of ligands related to the receptor. Moreover, ligand-receptor binding free energy is another factor for judgment. Though it is not entirely clear how to obtain specific bioactivity values from the association constants (Ka) and dissociation constants (Kd), the binding energy produced out of molecular docking is directly related to bioactivity values (92). This issue is compensated by molecular dynamics simulation. Based on a quantum chemical approach, molecular dynamics simulation explicitly deals with all or some of the electrons at the quantum mechanical level, thereby determining energies and molecular geometries of high precision. Molecular dynamics simulation of the ligand-receptor relationship is the last hurdle in the screening process (93). Available tools are Auto Dock, Dockey, Openeye, Mosoft, etc. (94). Gromacs and VMD are also used in molecular dynamics simulation (95).

Angiotensin-converting enzyme 2 is one of the most commonly used targets in SARS-CoV-2 inhibitory peptide studies. ACE2 causes blood pressure to drop (96). SARS-CoV-2 infection requires the binding of S protein to ACE2 in the cell membrane. ACE2 acts as a cellular receptor for viral entry (97). Cao et al. (98) used human ACE2 residues, rotamer interaction field docking, and large *in silico* miniprotein libraries to designed LCB1 and LCB3. They characterized the LCB1-LCB3 binding structure and the RBD-LCB3 binding structure by cryo-electron microscopy. And the force of action of LCB1 and LCB3 is similar to that of ACE2. The IC_50_ of LCB1 and LCB3 against SARS-CoV-2 was 23.540 and 48.100 pM, respectively. LCB1 and LCB3 were found to be six times more effective against SARS-CoV-2 than common antibodies. Miniature protein conjugates can be used to produce spray and aerosol agents, which can be added to existing delivery methods. Gouda et al. (99) and Basit et al. (100) designed an ACE2-derived peptide that binds well to SARS-CoV-2 in molecular docking, with a binding energy of 154.5 and 13.2 kcal/mol, respectively. Based on the human ACE2 sequence and the RBD region of the S protein of SARS-CoV-2 or SARS-CoV, Chen et al. (101) created a series of peptides, two of which performed well: AYp28 (KKKKKKKVEGFNCYFPLQS) and AYn1 (KKKKKKDKFNHEAEDLFY). In their computational simulations of peptide-protein interactions, the two peptides demonstrated a strong binding affinity to S protein and ACE2. The AYp28 peptide was shown to bind to ACE2 in cellular experiments, which resulted in a drop in intracellular levels of markers such as TMPRSS. Experiments on animals have also shown that AYp28 and AYn1 can inhibit viral invasion. These four studies suggest that ACE2-derived peptide mimics are an established approach against SARS-CoV-2 and provide new opportunities for COVID-19 treatment.

M^pro^ is one of the key enzymes associated with viral gene expression and replication, making it a target of anti-SARS-CoV-2 drugs (102). Hemmati and Tabein (103) obtained two peptides as potent agents: RKGCPPH derived from the desert locust, and NPCACFRNY derived from the assassin bug. They bind more tightly and more stably to M^Pro^. The binding sites of the two antiviral peptidesto M^Pro^ are similar to those of lopinavir and ritonavir, two effective anti-SARS-CoV-2 medications. It was reported that four SARS-CoV-2 inhibitory peptides derived from antimicrobial peptides bind to Domain 2 and Domain 3 of SARS-CoV-2 M^Pro^ (104). These findings suggest that antiviral peptides targeting M^pro^ can also be screened using molecular docking and molecular dynamics simulation.

The genes encoding the 5′ proximal two-thirds of the SARS-CoV-2 RNA genome are translated into pp1a and pp1ab. These polyproteins are cleaved by M^Pro^ and PL^Pro^ to form 16 non-structural proteins (Nsp1-16) (105), Nsps are important for viral replication. Alomair et al. (106) used neural networks and molecular dynamics simulation to predict the effect of phosphorylation on Nsps. Root mean square fluctuation values showed that phosphorylation has an impact on Nsp1-6 and Nsp16. Residues Gly281, Lys430, Pro434, and Phy 437 on Nps13 structural domain 1A and Tyr457, Asp458, and Lys460 on Nps13 structural domain 2A were identified by Raubenolt et al. (107) as potential active sites for the Nps13 binding pocket. Finding these Nsps active sites makes it easier to find and study SARS-CoV-2 inhibitory peptides against Nsps targets in the future. Currently, there are a limited number of studies on animal protein peptides related to M^Pro^ and Nsps; more articles focus on the mechanism of Nsps in the process of SRAS-CoV-2 invasion into body. Nsps inhibitory peptides will become a major hotspot in the field of bioactive peptide research. With the advancement of technology, the size and diversity of data have increased dramatically, and more convenient and accurate research methods are imminent. The emergence of bioinformatics has expanded the database and laid the foundation for computer-aided drug design. The development of AI has continued to advance, contributing to the development of computer-aided drug design. Several articles have mentioned the use of AI in combination with molecular docking, molecular dynamics simulation, and QASR to improve the speed and quality of SARS-CoV-2 inhibitory peptides screening.

Computer-aided drug design offers a time-saving alternative to conventional techniques for research on antiviral peptides. With scientific and technological advancements, it is expected to be increasingly applied in experimental research. Nevertheless, researchers should possess specialized expertise and adhere to rigorous experimental designs, so as to validate the outcomes obtained through bioinformatics approaches. Notably, the assessment of novel potential drugs should not solely rely on simulation results. Bioinformatics approaches are capable of effective antiviral peptide screening, clarifying the mechanism of action, and intelligently designing food-derived peptides suitable for nutraceutical, cosmeceutical, and pharmaceutical use.

## 6 Phage display

Phage display was invented in 1981 (108). It is a type of genetic modification technique, in which genetic modification of phages enables the target gene to bind to the phage shell protein, so that the corresponding fragment can be expressed on the phage surface when the phage is infected (109). Researchers found that phages have several advantages. For example, phage genome can accommodate larger exogenous DNA fragments; the transformation of phage is high; phage genome is convenient for DNA recombination and genetic manipulation; the expression level of phage fusion proteins is easy to control and regulate; and phages are genetically stable (110). With the development and wide application of phage display, a phage display library has been established, paving the way for subsequent research and experiments. These advantages make phage display a widely used tool in bioengineering research, such as screening of ligands, development of new drugs, vaccine design, disease diagnosis, and delivery of targeted drugs (111). Since the COVID-19 outbreak, researchers have been actively searching for anti-SARS-CoV-2 vaccines. Phages play an important role in related studies. Gaynor et al. (112) utilized phage display library screening to discover small-molecule inhibitors. They found a multivalent bicyclic peptide that acts on the S protein of SARS-CoV-2. A multivalent bicyclic peptide is a thioether-bonded bicyclic peptide formed by three cysteines that are cyclized *in situ* pairing with an amido-PEG10-triazolyl linker. Multivalent bicyclic peptides inhibit SARS-CoV-2 replication and cell–cell fusion, as demonstrated in animal studies. Pang et al. (113). used a phage display approach that combined non-amplification panning and single-amplification panning to identify the phage displaying the cyclic heptapeptide ACLDWLFNSC. This phage binds specifically to the S protein of SARS-CoV-2 and is highly stable.

Phage display can reveal the linkage between a molecule and the DNA sequence encoding the gene of the displayed molecule, and determine the amino acid sequence of the binding agent produced by the inserted fragment. These advantages contribute to the application of phage display in various fields, ranging from material utilization to diagnostics to the development of novel therapeutic drugs.

## 7 Drug delivery methods for SARS-CoV-2 inhibitory peptides from animal protein sources

Animal protein-derived SARS-CoV-2 inhibitory peptides have anti-COVID-19 effects. However, some drawbacks remain. Animal protein-derived SARS-CoV-2 inhibitory peptides have a short half-life. This is mostly due to the fact that enzymes can easily hydrolyze peptides (114). Enzymes in the oral cavity and gastrointestinal tract readily break down bioactive peptides when they are administered orally (115). The drug distribution method makes up for this deficiency. Bioactive peptides can be delivered using a range of techniques, including intramuscular, subcutaneous, and intravenous injections (116). However, these delivery routes cannot guarantee that the bioactive peptides can reach the designated site. Therefore, various carriers have been created to facilitate the effective delivery of bioactive peptides. The drug delivery system based on those carriers can reduce the side effect on healthy cells, increase stability, extend the half-life of bioactive peptides, maintain a steady and effective blood concentration of bioactive peptides, and guarantee that the drug is concentrated in the target tissue (117).

An artificial cell membrane polymer is a self-assembling nanoscale vesicle that can be efficiently absorbed by dendritic cells. Containing medications and antibodies, an artificial cell membrane polymer enables delivery to certain tissue targets (118). The medication in a polymer induces the body to modify the immune response, so as to provide the desired immunological impact when it is absorbed by the dendritic cells. Polypeptide vaccines are a good fit for this delivery method. In addition, there are other novel carriers. Biodegradable polymer micro-cells are stable, biocompatible, non-toxic, biodegradable, and injectable. Also, they can continuously provide encapsulated antigen by delaying its release (119). However, the organic compounds used to create biodegradable mono-polymer microspheres may have an adverse effect on the activity of bioactive peptides. In pharmacodynamic simulations and cell experiments for SARS-CoV-2 infection, supermolecular polymer nanoparticle hydrogel have been found to perform well. It can enhance the stability and biocompatibility of protein medications in the body (120). However, the hydrogel and the cell have an erratic osmotic relation, thereby degrading the protein and releasing the enclosed medicine. Lungs are most severely affected by SARS-CoV-2, primarily due to respiratory infection. Additionally, there is a novel method of preventing the virus from entering the lungs through the nose. LCB1, which is a SARS-CoV-2 inhibitor, can bond with trehalose and L-leucine to form ultra-thin particles. This mode of delivery enables SARS-CoV-2 inhibitory peptides to directly act on target organs without destroying the polymeric structure, contributing to the development of potential anti-SARS-CoV-2 drugs (121). It has been demonstrated that LCB1 peptides in dry powder form are effective in fighting the SARS-CoV-2 Delta variant but ineffective in combating the Omicron strain.

The effectiveness of the delivery of SARS-CoV-2 inhibitory peptides from animal protein sources is significantly influenced by the carrier’s stability and inclusivity. The application of bioactive peptides is further expanded by injectable vectors. However, different carriers have different drawbacks. The key to developing new drugs is to find appropriate carriers for those SARS-CoV-2 inhibitory peptides. Future research is likely to focus heavily on combining carriers with SARS-CoV-2 inhibitory peptides of animal origin.

## 8 Application prospects and challenges of SARS-CoV-2 inhibitory peptides from animal protein sources

Few papers published so far have focused on SARS-CoV-2 inhibitory peptides produced from animal proteins, leaving great room for future research on these peptides.

Researchers in the fields of health care and skin care have taken notice of the broad activity of animal protein-derived peptides (122). There is great potential for anti-SARS-CoV-2 pharmacological developments. The pharmaceutical industry is interested in further research on SARS-CoV-2 inhibitory peptides because of their non-toxicity, low cost, and targeting properties (123). SARS-CoV-2 inhibitory peptides derived from animal proteins are potential candidates for COVID-19 medications (15). Besides, the peptides’ nutrition and additional properties, including anti-inflammatory, antibacterial, and antioxidant properties, benefit patients’ quicker recovery, and dietary replenishment.

At the moment, milk, venom, and fish are the primary material sources of SARS-CoV-2 inhibitory peptides of animal origin. SARS-CoV-2 inhibitory peptides from milk sources are more effective and can prevent the main target from SARS-CoV-2 infection. The primary sites of SARS-CoV-2 infection in the human body should be studied in research on novel SARS-CoV-2 inhibitory peptides from animal protein sources. Additionally, more research should be conducted on the mechanism and activity of SARS-CoV-2 inhibitory peptides. The conventional approach to identifying SARS-CoV-2 inhibitory peptides from animal proteins is expensive and time-consuming. Phage display technology and computer-aided drug design can save time, but they still require experimental verification, and the validity of experimental outcomes should be considered (124). Peptide databases are the foundation of computer-aided drug discovery, and related research is based on increasing the quantity of peptides and expanding related data in these databases. Few articles have addressed the need for animal and clinical trials to confirm the safety of polypeptide-based agents in humans before incorporating SARS-CoV-2 inhibitory peptides of animal origin into drug therapies. The animal proteins used to create SARS-CoV-2 inhibitory peptides can be readily degraded by enzymes, which prevents polypeptides from accumulating in the human body and smoothly reaching the intended target position, affecting the medication’s impact. Encapsulated peptides not only show greater stability by adherence to carriers but also increases the efficiency of injectable delivery compared with the conventional oral administration method. Nevertheless, further experimental research is necessary to identify vectors that interact appropriately with polypeptides (125). The difficulties in the identification, preparation, delivery, and validation of SARS-CoV-2 inhibitory peptides of animal origin are yet to be solved.

## 9 Conclusion

This review includes a thorough analysis of the SARS-CoV-2 infection mechanism, the use of animal proteins as a raw material for the preparation of SARS-CoV-2 inhibitory peptides, computer-aided design, phage display technology, and drug administration methods. Multiple intervention targets that could be used to slow the spread of SARS-CoV-2 have been found by researchers, including Nsps, ACE2, and SARS-CoV-2 proteases like M^Pro^ and PL^Pro^. Nonetheless, the research on the relationship between Nsps and inhibitory peptides produced from animal proteins is insufficient.

Animal protein-derived SARS-CoV-2 inhibitory peptides can be prepared using fish, venom, and milk. It was found that milk has the greatest potential to produce SARS-CoV-2 inhibitory peptides. Using computer-aided drug design technologies, new SARS-CoV-2 inhibitory peptides of animal origin can be tested, and the relationship between SARS-CoV-2 and the inhibitory peptides can be examined. The combination of AI and QSAR analysis has great potential to accelerate the identification of novel SARS-CoV-2 inhibitory peptides. These techniques can theoretically facilitate the discovery of SARS-CoV-2 inhibitory peptides made from animal proteins. New peptides that can inhibit SARS-CoV-2 can be found using the phage display method. Drug delivery techniques contribute to effective *in vivo* distribution of SARS-CoV-2 inhibitory peptides derived from animal proteins. These techniques have allowed for continuous optimization and development of SARS-CoV-2 inhibitory peptides of animal origin, opening up new possibilities for COVID-19 research and development. These results suggest that peptides made from animal proteins have great potential for protecting human health. Peptide safety and efficacy are expected to be enhanced in the near future with the development of drug delivery techniques.

The paper puts forward some suggestions for future research on animal protein-derived SARS-CoV-2 inhibitory peptides: (1) The interaction between Nsps and SARS-CoV-2 inhibitory peptides of animal origin should be investigated. (2) The viability of combining different AI techniques with computer-aided drug design should be examined. (3) The phage display technology should be used to investigate SARS-CoV-2 inhibitory peptides from animal protein sources. (4) Appropriate vectors for animal protein-derived SARS-CoV-2 inhibitory peptides should be identified for drug development.

## Author contributions

XK: Writing – original draft, Writing – review & editing. WW: Writing – original draft, Writing – review & editing. YiZ: Writing – original draft, Writing – review & editing. NW: Writing – original draft, Writing – review & editing. KB: Writing – original draft, Writing – review & editing. YW: Writing – original draft, Writing – review & editing. QQ: Writing – original draft, Writing – review & editing. YuZ: Writing – original draft, Writing – review & editing. XL: Writing – original draft, Writing – review & editing. JX: Writing – original draft, Writing – review & editing.
